# A tripeptidyl peptidase 1 is a binding partner of the Golgi pH regulator (GPHR) in *Dictyostelium*

**DOI:** 10.1242/dmm.029280

**Published:** 2017-07-01

**Authors:** Maria Stumpf, Rolf Müller, Berthold Gaßen, Regina Wehrstedt, Petra Fey, Malte A. Karow, Ludwig Eichinger, Gernot Glöckner, Angelika A. Noegel

**Affiliations:** 1 Institute of Biochemistry I, Medical Faculty, University Hospital Cologne, Center for Molecular Medicine Cologne, University of Cologne, Joseph-Stelzmann-Str. 52, Köln 50931, Germany; 2 Dicty Base, Northwestern University, Biomedical Informatics Center and Center for Genetic Medicine, Chicago, IL 60611, USA

**Keywords:** Neuronal ceroid lipofuscinosis (NCL), Tripeptidyl peptidase 1 (TPP1), Golgi pH regulator (GPHR), Endosomes, Lysosomes

## Abstract

Mutations in tripeptidyl peptidase 1 (*TPP1*) have been associated with late infantile neuronal ceroid lipofuscinosis (NCL), a neurodegenerative disorder. TPP1 is a lysosomal serine protease, which removes tripeptides from the N-terminus of proteins and is composed of an N-terminal prodomain and a catalytic domain. It is conserved in mammals, amphibians, fish and the amoeba *Dictyostelium discoideum*.* D. discoideum* harbors at least six genes encoding TPP1, *tpp1A* to *tpp1F*. We identified TPP1F as binding partner of *Dictyostelium* GPHR (Golgi pH regulator), which is an evolutionarily highly conserved intracellular transmembrane protein. A region encompassing the DUF3735 (GPHR_N) domain of GPHR was responsible for the interaction. In TPP1F, the binding site is located in the prodomain of the protein. The *tpp1F* gene is transcribed throughout development and translated into a polypeptide of ∼65 kDa. TPP1 activity was demonstrated for TPP1F-GFP immunoprecipitated from *D. discoideum* cells. Its activity could be inhibited by addition of the recombinant DUF3735 domain of GPHR. Knockout *tpp1F* mutants did not display any particular phenotype, and TPP1 activity was not abrogated, presumably because *tpp1B* compensates as it has the highest expression level of all the *TPP1* genes during growth. The GPHR interaction was not restricted to TPP1F but occurred also with TPP1B. As previous reports show that the majority of the TPP1 mutations in NCL resulted in reduction or loss of enzyme activity, we suggest that *Dicyostelium* could be used as a model system in which to test new reagents that could affect the activity of the protein and ameliorate the disease.

## Abstract (precis)

**Summary:** Interaction of *Dictyostelium* tripeptidyl peptidase 1 with GPHR could be relevant for studies of the human enzyme, which is associated with a neurodegenerative disorder.

## INTRODUCTION

Tripeptidyl peptidase 1 (TPP1) enzymes are lysosomal peptidases that have a tripeptidyl exopeptidase activity with an optimal pH of 4-5. They belong to the group of sedolisins, serine peptidases that are present in organisms ranging from bacteria to mammals (Wlodawer et al., 2001; Comellas-Bigler et al., 2002). In eukaryotes, they are synthesized as precursors with an N-terminal signal sequence, a prodomain and a catalytic domain (peptidases S53 domain) (Vines and Warburton, 1999). Furthermore, they are N-glycosylated and become mannosylated during passage through the Golgi complex (Kollmann et al., 2013). TPP1 removes tripeptides from the N-terminus of proteins, but the *in vivo* substrates are not well characterized. However, synthetic peptides have been developed for enzyme analysis (Tian et al., 2006).

In humans, TPP1 is encoded by the *TPP1* (*CLN2*) gene. Mutations in this gene cause an autosomal recessive neurodegenerative disease of childhood called late infantile neuronal ceroid lipofuscinosis (NCL). NCL is not only caused by *TPP1* mutations but can result from mutations in several other genes. So far, 13 loci, CLN1 to CLN14 (CLN9 has not yet been identified), have been reported, which encode proteins with different activities ranging from protease to chaperone functions (http://www.ucl.ac.uk/ncl/mutation.shtml). Although the exact function of many of the proteins is not known, in all cases the diseases associated with their malfunctioning are considered lysosomal storage diseases (Kollmann et al., 2013). NCL associated with *TPP1* mutations (OMIM 204500) leads to premature death of the affected individuals (Cárcel-Trullols et al., 2015). The mutations identified in *TPP1* (http://www.ucl.ac.uk/ncl/CLN2mutationtable.htm) are spread over the whole protein, and the majority result in reduction or loss of enzyme activity (Wlodawer et al., 2003; Kousi et al., 2012). CLN2 has been linked to macroautophagy, based on the observed impaired formation of autophagosomes in patient fibroblasts carrying *TPP1* mutations, and to alterations in endosomal-lysosomal cell processes (Micsenyi et al., 2013; Vidal-Donet et al., 2013).

A tripeptidyl peptidase 1 (ddTPP1, TPP1A, DDB_G0269914) has been identified previously in the single-celled amoeba *Dictyostelium discoideum*. Upon starvation, *D. discoideum* cells chemotactically aggregate and form a multicellular organism, in which the cells differentiate into different cell types. Finally, a fruiting body consisting of a base, a stalk and a head containing spores is formed. The spores germinate under appropriate conditions and the amoebae initiate a new life cycle (Huber, 2016).

Transcription of the *tpp1A* gene is developmentally regulated. No transcripts are detected in vegetative cells, but they accumulate during development and reach a maximum at late developmental stages. Overall, the transcripts were found to be not very abundant when examined by RNA-seq (Parikh et al., 2010; Schilde et al., 2016). Disruption of the gene causes developmental defects: in particular, spore formation was defective, and culture under autophagy-inducing conditions left the *tpp1A* mutant cells less viable than control cells. GFP-tagged TPP1A was found to be localized to some but not all lysosomes (Phillips and Gomer, 2015).

We identified another TPP1, TPP1F (DDB_G0281823), as an interaction partner of *D. discoideum* GPHR in pull-down experiments. GPHR is a conserved protein present in plants and animals. In humans, it is encoded by two genes, *GPR89A* and *GPR89B*. GPHR is a transmembrane protein characterized by a DUF3735 domain (domain of unknown function)/GPHR_N and a C-terminal ABA_GPCR superfamily domain. The DUF3735 domain is found in all eukaryotes and is ∼60-70 amino acids in length. It consists of a transmembrane domain and its neighboring sequences. In GPHR, it comprises the loop after the fourth transmembrane domain, the fifth transmembrane domain and adjacent loop sequences (Deckstein et al., 2015). The ABA_GPCR domain received its name from the abscisic acid (ABA) G-protein coupled receptor (GPCR) found in plants and which has been proposed to act as a receptor for this hormone (Pandey et al., 2009). Initially, this family was known as orphan G protein-coupled receptor 89 (GPR89) class. More recent research indicated that it may not belong to the GPCRs since the proteins do not have the characteristic seven transmembrane domain structure typical of GPCRs. Instead, eight or nine transmembrane domains are predicted (Taddese et al., 2014).

The proposed functions of the GPHR/GPR89 proteins vary widely. However, the initially reported role of the plant proteins GTG1 and GTG2 as ABA receptors has been questioned, and a more recent report proposed that GTG1 is primarily located at the Golgi and the endoplasmic reticulum (ER) and is important for cell growth and development (Jaffé et al., 2012). The mammalian protein was identified as a Golgi pH regulator. HA-tagged protein expressed in CHO cells colocalized with Golgi proteins and showed some reticular pattern reminiscent of the ER. It did not colocalize with lysosomal or endosomal markers (Maeda et al., 2008). For mouse, a keratinocyte-specific GPHR knockout is available. Mutant mice had a skin barrier defect, which was proposed to result from the formation of aberrant lamellar granules that originate from the Golgi and contain lipids, proteases and proteins required for formation of the skin barrier (Tarutani et al., 2012). In *Drosophila*, complete inactivation of the gene was not always lethal. Instead, the mutants had aberrant Golgi and ER structures. They could go through embryonic development, but most of them died at late larval stages (Charroux and Royet, 2014). The *Dictyostelium* protein is present in the ER and in Golgi membranes. Mutant cells have growth and developmental defects, and processes related to membrane trafficking such as yeast particle uptake and mannosidase secretion are impaired (Deckstein et al., 2015).

Here, we identified TPP1F as a GPHR binding partner, studied the interaction and analyzed TPP1F in more detail. TPP1F transcripts are more abundant than those of TPP1A and are present in the growth phase and throughout development. We determined the localization of the protein and compared it with that of GPHR using monoclonal antibodies against TPP1F and GFP-tagged proteins. TPP1F localized to the ER and to cytoplasmic structures decorated by antibodies specific for the vacuolar ATPase (V-ATPase), which represent the endo-lysosomal system. We report further that GPHR is not only present at the ER and Golgi but also at the V-ATPase-positive compartment. The interaction between TPP1F and GPHR appears to be mediated by the DUF3735 domain of GPHR and the prodomain of TPP1F and might affect the activity of the enzyme. We describe the TPP1 family in *D. discoideum* and show that the interaction with GPHR is not restricted to TPP1F but is also observed for TPP1B, which shows the highest transcript levels for all TPP1 enzymes of *D. discoideum*. Cells lacking TPP1F did not exhibit obvious defects in growth and development, which might be due to other TPP1 proteins taking over the functions.

## RESULTS

### Identification of TPP1F as a binding partner of GPHR

**Fig. 1.**
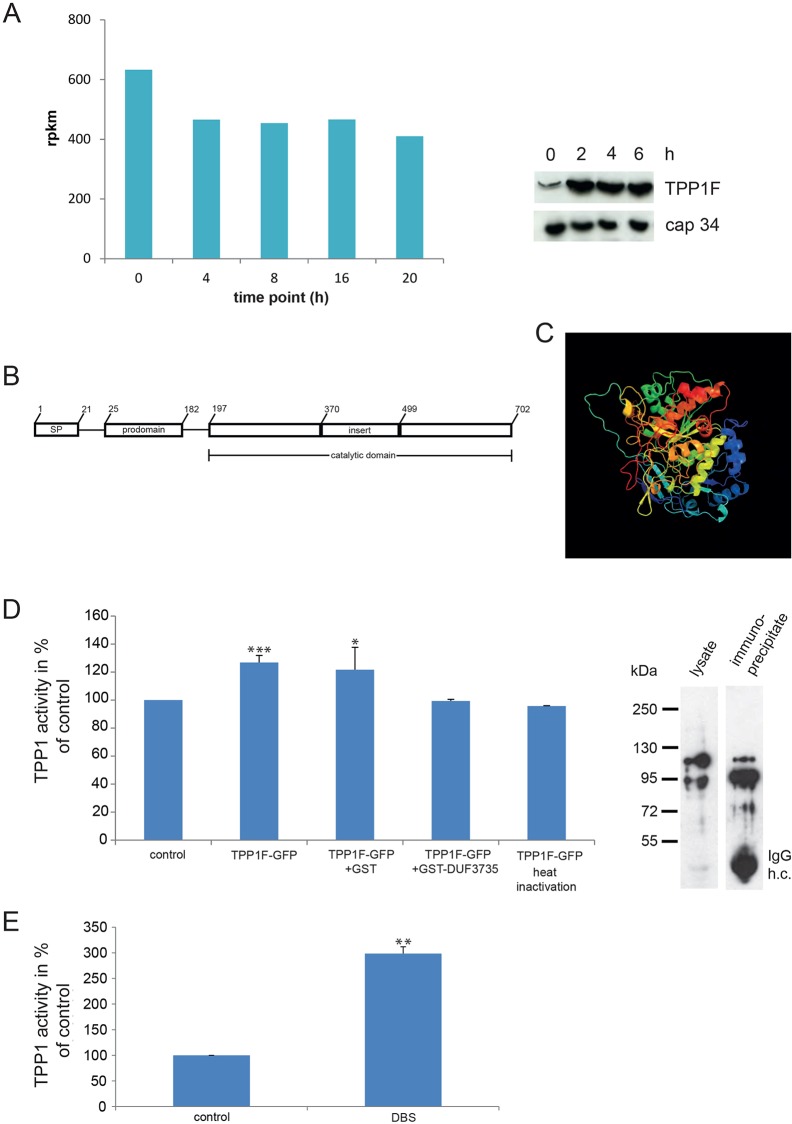
**Characterization of TPPF1.** (A) *tpp1F* transcript accumulation during development (left). The data were obtained from RNA-seq experiments as described in the Materials and Methods. 0 h time point corresponds to the growth phase, 4 and 8 h correspond to aggregation stages, 16 h corresponds to the slug stage and 20 h to the culmination phase. Rpkm, reads per kb per million mapped reads. Right panel, TPP1F protein level during early development. AX2 cells were starved in suspension for the indicated time points and whole-cell lysates prepared. 4×10^5^ cell equivalents were loaded per lane. The blot was probed with mAb K88-7-2 for TPP1F (65 kDa) and mAb 135-409 recognizing cap34 (34 kDa) as a loading control. (B) Domain structure of TPP1F as predicted with the NCBI conserved domain database (CDD) (Marchler-Bauer et al., 2015). SP, signal peptide. (C) Three-dimensional structure of TPP1F as calculated by the Phyre^2^ program (Kelley et al., 2015). (D) Tripeptidyl peptidase activity of TPP1F. TPP1F-GFP was immunoprecipitated from AX2 transformants and the precipitate used for the assay. The impact of GST-DUF3735 and GST for control on the activity was also tested as well as heat treatment. The fluorescence reading of the control (buffer and substrate) was set to 100%. The values represent the mean±s.d. of three to five experiments. *P≤0.05, ***P≤0.001, in comparison to control. Right panel shows western blot of the protein precipitated with polyclonal antibodies and used in the assay. The blot was probed with mAb K3-184-2. The band at ∼55 kDa corresponds to the heavy chain (h.c.) of the anti-GFP pAb. (E) TPP1 activity in mouse blood (dried blood spot, DBS). The control represents the fluorescence value of a filter paper in buffer containing the reagent and was set to 100%. The values are the mean of two experiments. *P*≤0.01.

To identify binding partners of *D. discoideum* GPHR we used cell lysates of GPHR-knockdown cells expressing GPHR-GFP and carried out immunoprecipitation experiments with polyclonal anti-GFP antibodies (Deckstein et al., 2015). As a control, AX2 cells expressing GFP were used. In the immunoprecipitate with GPHR-GFP we identified three components of the V-ATPase, the V-ATPase subunits A, D and M, ribosomal proteins, different enzymes and the protein encoded by the *v4-7* gene (for the full list see Table S1). These proteins were not present in the control experiment. The interaction with ribosomal proteins was considered unspecific and interpreted as representing a charge interaction between the basic ribosomal proteins and the more acidic *v4-7* gene product (see below). The *v4-7* gene (DDB_G0281823) located on chromosome 3 received its name from vegetative stage-specific mRNA 4-7. Data from RNA-seq experiments show that its transcripts are very abundant throughout development and are particularly high at the vegetative stage (Fig. 1A, left panel). We also probed western blots containing cell homogenates obtained from AX2 cells grown in shaken suspension with monoclonal antibodies recognizing the *v4-7* gene product, now called TPP1F (see below), and observed high levels at every time point (Fig. 1A, right panel). The amounts of the protein were, however, lower during growth (t0) than during development. A discrepancy between mRNA and protein levels is not uncommon, and this phenomenon has been discussed extensively in recent years (de Sousa Abreu et al., 2009; Vogel and Marcotte, 2012). There can be many reasons for this, such as inhibition of translation of the mRNA.

**Fig. 2.**
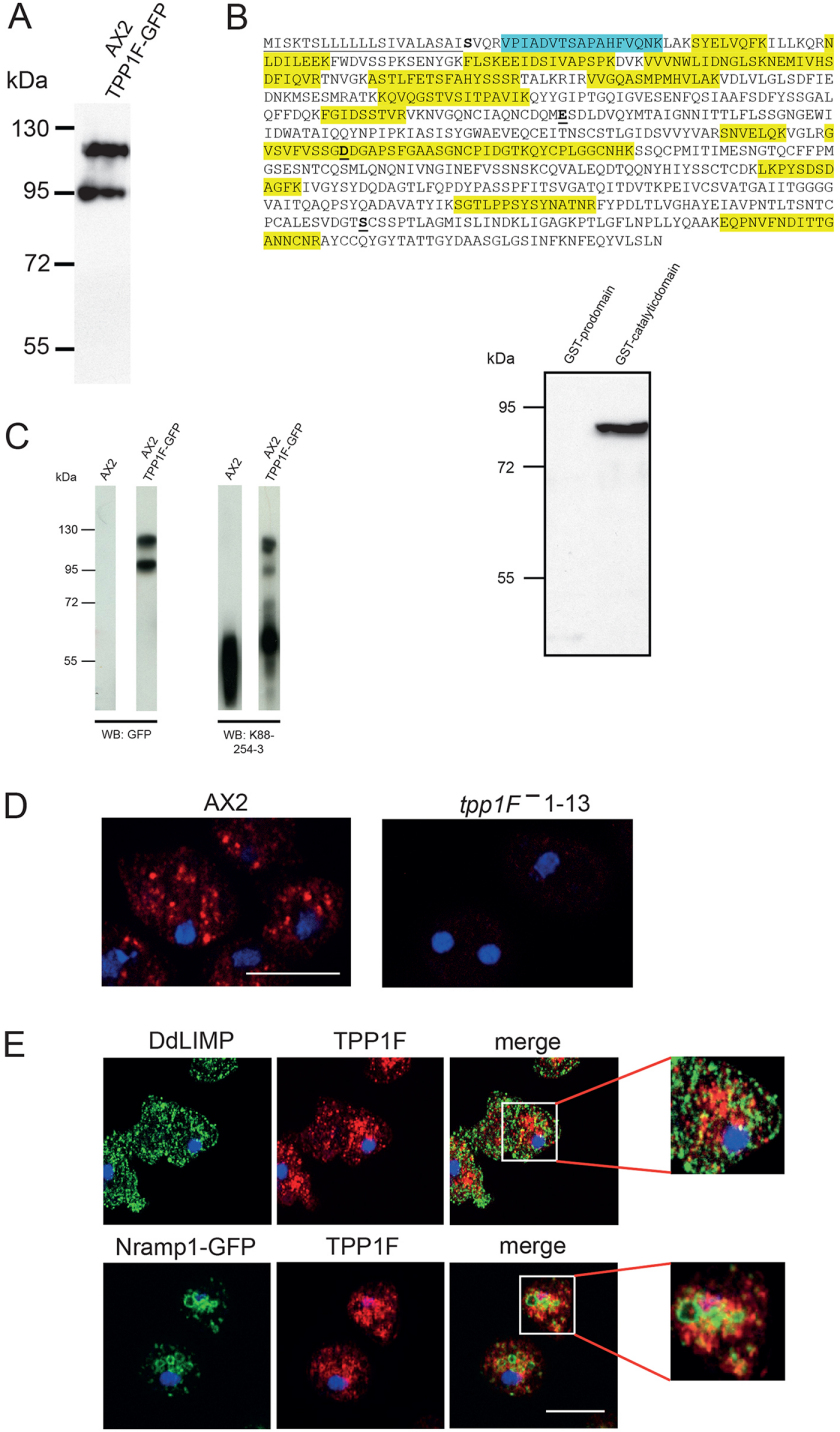
**Characterization of TPP1F-GFP and TPP1F-specific antibodies.** (A) TPP1F-GFP expression in AX2. Proteins from whole-cell lysate (corresponding to 4×10^5^ cell equivalents) were separated by SDS-PAGE (10%) and probed with mAb K3-184-2. (B) Amino acid sequence of TPP1F. Underlined is the N-terminal signal sequence, in bold and underlined are the catalytic residues E272, D369 and S611. Highlighted in yellow are the peptides that were identified by mass spectrometry analysis in the two polypeptides immunoprecipitated by mAb K3-184-2. The peptide in turquoise was present only in the higher molecular mass band. (C) Left panel shows whole-cell lysates from AX2 and AX2 expressing TPP1F-GFP probed with GFP-specific mAb K3-184-2 and TPP1F-specific mAb K88-254-3. Right panel, mAb K88-254-3 specifically recognizes the catalytic domain but not the prodomain expressed as GST-fusion proteins. (D) Immunofluorescence analysis of AX2 and TPP1F-deficient cells (clone 1-13). mAb K88-254-3 was used for labeling. DAPI staining of nuclei is shown in blue. Cy3-labeled secondary antibodies were used for detection. (E) Localization of TPP1F, DdLIMP and Nramp1. AX2 cells stained with mAb K88-254-3 for TPP1F and polyclonal DdLimp-specific antibodies. AX2 cells expressing Nramp1-GFP are labeled with K88-254-3. Nuclei are stained with DAPI. Scale bars: 10 µm.

The *v4-7* gene encodes a protein of 702 amino acids, TPP1F, with a predicted molecular mass of 75.78 kDa and a pI of 5.04. TPP1F contains an N-terminal hydrophobic sequence, which is predicted as a signal sequence by SignalP 4.1 (http://www.cbs.dtu.dk/services/SignalP). According to the prediction, the cleavage occurs between residues 21 and 22. A database search revealed high homology to tripeptidyl peptidase 1 (TPP) from various organisms. Like other tripeptidyl peptidases, the *D. discoideum* protein has a predicted prodomain (residues 25-182) and a catalytic domain (peptidases S53 domain), which extends from residue 197 to the end. In TPP1F, the catalytic domain is interrupted by a stretch of amino acids (residues 370-499), for which no structure prediction was available. The catalytic triad, a particular sequence of amino acids (S611, E272, D369 in TPP1F), which represent the catalytic residues and are highly conserved in the S53 sedolisin family of peptidases, to which TPP1 proteins belong, and a Ca^2+^ binding site are also present (Fig. 1B, see also Fig. 2B). Overall, the *Dictyostelium* and human proteins exhibit 26.0% identity and 51.7% similarity at the amino acid level. Using the Phyre^2^ (Protein Homology/analogY Recognition Engine v.2.0) secondary structure and disorder prediction program, 530 residues (75% of the sequence) have been modeled with 100% confidence by the single highest-scoring template c3edyA (Chain A; PDB molecule: tripeptidyl peptidase 1; title: crystal structure of the precursor form of human2 tripeptidyl peptidase 1) revealing a tripeptidyl peptidase 1 structure (Fig. 1C) (Kelley et al., 2015; Guhaniyogi et al., 2009; Pal et al., 2009). In contrast to mammalian TPP1, *Dictyostelium* TPP1F does not harbor potential N-glycosylation sites.

We next investigated whether TPP1F indeed has tripeptidyl peptidase activity. For this, we expressed the protein as a GFP-tagged fusion (TPP1F-GFP, amino acid residues 1-702) in *D. discoideum* strain AX2. The GFP tag was added to the C-terminus because the N-terminus of TPP1F corresponded to a signal sequence, which is essential for transport of the protein across the ER membrane and is presumably cleaved off. The protein was immunoprecipitated with polyclonal anti-GFP antibodies and the immunoprecipitate used in TPP1 assays (Fig. 1D, right panel). We observed that the protein could cleave Ala-Ala-Phe-7-amido-4-methylcoumarin, a synthetic fluorescent substrate (Page et al., 1993), leading to an enhanced fluorescence. The activity was abrogated upon heat treatment. As a positive control for the enzymatic assay we used dried blood spots (DBSs) containing mouse blood (Fig. 1E). GFP antibodies bound to beads, which were used as a control, did not influence fluorescence (Fig. 1D).

Upon analysis of TPP1F-GFP in western blots, we identified two proteins of ∼95 and 120 kDa with GFP-specific antibodies (Fig. 1D; Fig. 2A). To prove that the observed bands corresponded to the protein encoded by the *tpp1F* gene, we immunoprecipitated TPP1F-GFP with anti-GFP antibodies, followed by separation by SDS-PAGE and staining with Coomassie Blue. Bands at around 95 and 120 kDa were cut out and the proteins subjected to mass spectrometry analysis (LCMS). This resulted in the identification of TPP1F peptides in both bands. We identified 14 peptides that were exclusively derived from TPP1F and that were distributed over the whole protein, and almost all were present in both proteins (highlighted in yellow in Fig. 2B). The peptide closest to the N-terminus started at position 26 and was present only in the higher molecular mass band (Fig. 2B, highlighted in turquoise). Interestingly, this position is nearly identical with the predicted start of the prodomain (residue 25). The theoretical molecular size of TPP1F-GFP is ∼105 kDa. Since mammalian TPP1 is glycosylated it could be that the higher one of the bands observed in western blots is a glycosylated form. As mentioned above, TPP1F does not have N-glycosylation sites, however, Ser95 and Ser201 were predicted as potential O-glycosylation sites by the DictyOGlyc 1.1 server (www.cbs.dtu.dk/services/DictyOGlyc).

We also generated several monoclonal antibodies (mAbs) against recombinant GST-TPP1F (residues 20-702). They were initially identified based on their ability to recognize TPP1F-GFP in western blots. In whole-cell homogenates of AX2 cells, the antibodies recognized a single band with an apparent molecular size of ∼65 kDa (shown for mAb K88-254-3), which is smaller than the theoretical value (∼75 kDa) and could reflect a post-translational processing as was observed for the mammalian protein (Pal et al., 2009) (Fig. 2C, left panel; see also Fig. 1A). All antibodies reacted with the GST-tagged catalytic domain (residues 201-702) at ∼83 kDa, whereas the ∼50 kDa protein encompassing the prodomain domain (residues 21-200) was not recognized (Fig. 2C, right panel). In immunofluorescence analysis, the antibodies recognized dot-like structures in the cytoplasm as well as larger and smaller vesicular structures. In knockout mutants, the TPP1F-specific staining was abolished (see below) (Fig. 2D). In co-immunofluorescence experiments with TPP1F and anti-DdLIMP antibodies, we occasionally saw an overlap (Fig. 2E). DdLIMP is a homolog of mammalian CD36/LIMP-II, a lysosomal integral membrane protein. In *D. discoideum*, DdLIMP antibodies decorate only a subset of lysosomal vesicles, which presumably represent the cysteine protease-containing lysosome population (Karakesisoglou et al., 1999). When we detected GFP-tagged Nramp1, a component of the endosomal/lysosomal compartment (Sultana et al., 2005), along with TPP1F (using mAb K88-254-3) in AX2 cells, the overlap was also limited (Fig. 2E).

### Characterization of TPP1F-GFP

**Fig. 3.**
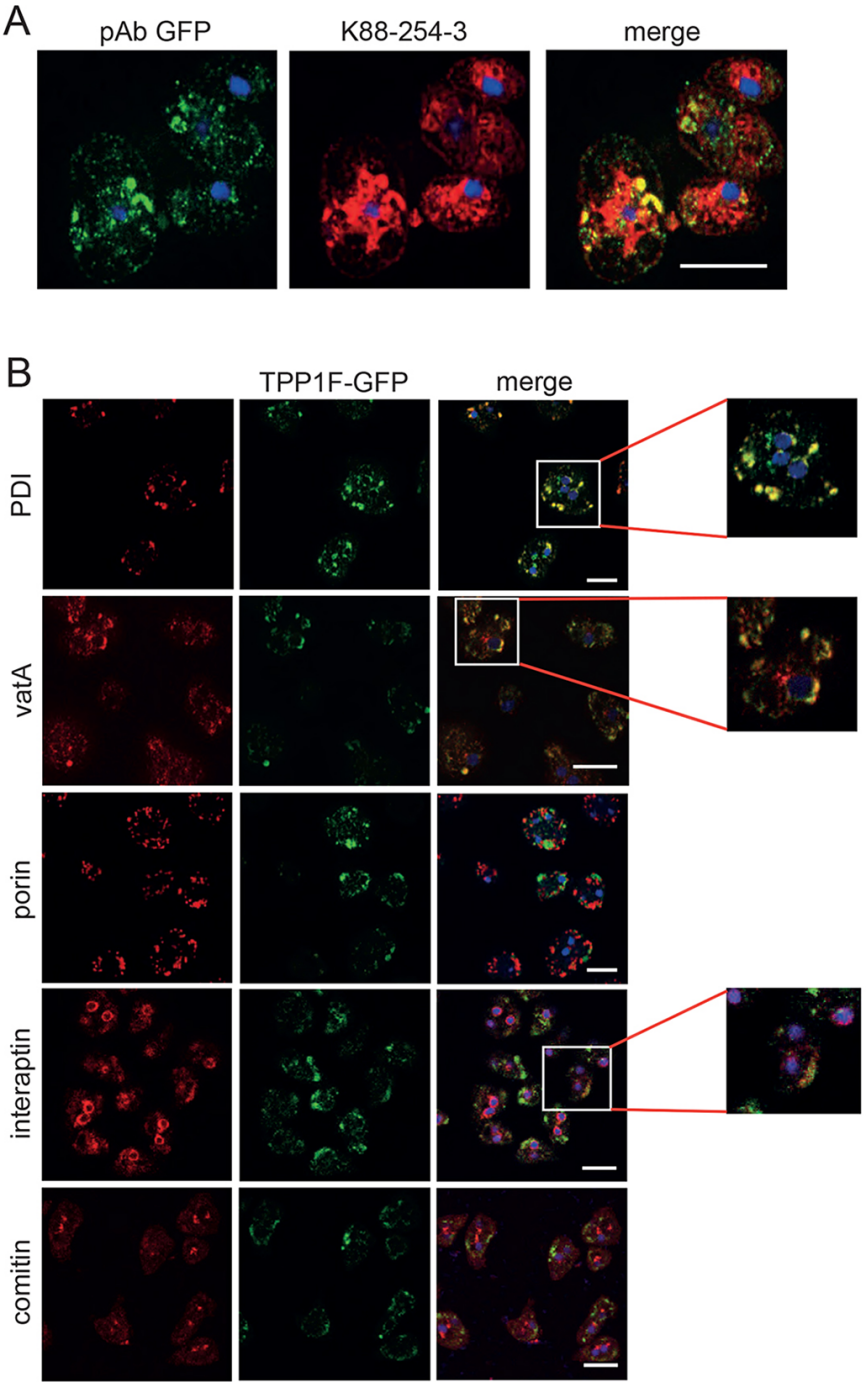
**Subcellular localization of TPP1F.** (A) Detection of TPP1F-GFP by mAb K88-254-3. TPP1F-GFP was detected with pAb GFP. Cells were fixed with methanol. (B) Distribution of TPP1F-GFP in AX2 compared with various marker proteins. Detection of TPP1-GFP was with polyclonal GFP-specific antibodies. Co-staining was with monoclonal antibodies detecting PDI (ER marker), vatA (endo-lysosomal membranes and membranes of the contractile vacuole), porin (mitochondria), interaptin (nuclear envelope and ER) and comitin (Golgi), as indicated. Nuclei are stained with DAPI (merge, in blue). Scale bars: 10 µm.

To determine the subcellular localization of TPP1F more precisely, we used TPP1F-GFP-expressing AX2 cells since they allowed us to use the battery of monoclonal antibodies available for *D. discoideum* proteins characteristic for particular subcellular compartments (Deckstein et al., 2015). When we initially analyzed the cells by live cell microscopy, we did not observe GFP fluorescence in the transformants despite the presence of GFP-positive proteins in western blots (see Fig. 2A). We then used polyclonal GFP-specific antibodies to detect TPP1F-GFP in fixed cells by immunofluorescence analysis. We observed dotted and occasionally vesicular staining. In co-immunofluorescence analysis, it partially overlapped with the mAb K88-254-3 staining (Fig. 3A). Furthermore, we carried out co-immunofluorescence studies with PDI-specific mAb 221-64-1 (Monnat et al., 1997) and vatA-specific mAb 221-35-2 (Jenne et al., 1998), and detected a partial colocalization in both cases. PDI (protein disulfide isomerase) is an ER marker and vatA is a subunit of the V-ATPase. The presence of TPP1F in the ER could be due to an interaction with its binding partner GPHR, which is also found in the ER (Deckstein et al., 2015). The partial colocalization with vatA-specific membranes might indicate TPP1F localization in specific subcompartments only. In comparison to endogenous TPP1F, the GFP-tagged version was expressed in significantly lower amounts, which makes inaccurate localization due to overexpression very unlikely (Fig. 2C).

No overlap was seen with mitochondria detected with porin-specific mAb 70-100-1 (Troll et al., 1992) and with the Golgi detected with mAb 190-340-8 against comitin (Weiner et al., 1993). Antibodies specific for interaptin, a protein of the nuclear membrane and also located at ER membranes, showed some colocalization of both proteins in cytoplasmic areas presumed to be ER (Rivero et al., 1998) (Fig. 3B). The fluorescence signal of EGFP in living cells depends on the organellar pH and is quenched in the acidic conditions of the lysosomes (pH <5). Therefore, it can only be detected by indirect immunofluorescence. This might explain our observations for TPP1F-GFP and supports its presence in the lysosome (Katayama et al., 2008).

### Analysis of the GPHR-TPP1F interaction

**Fig. 4.**
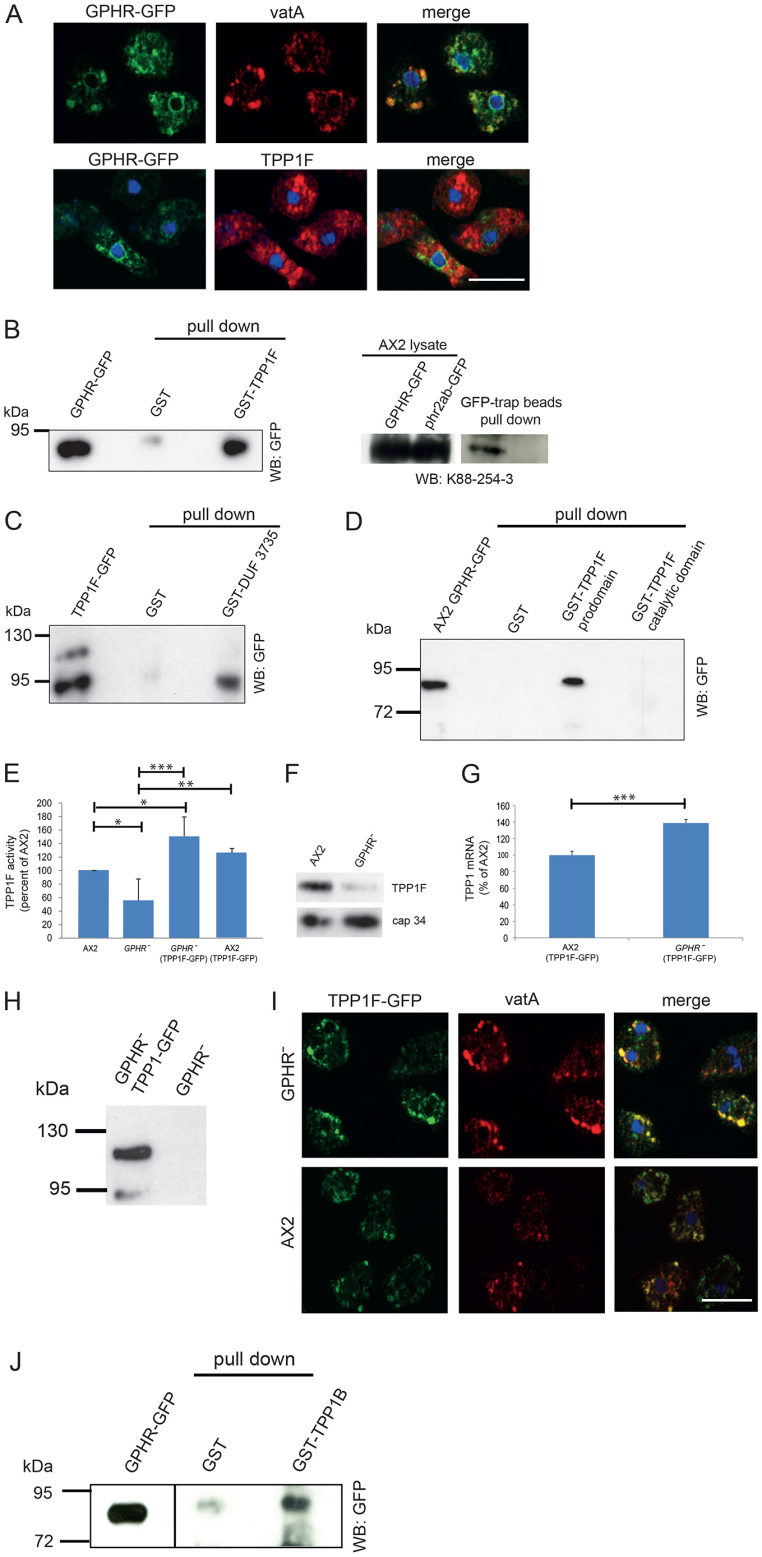
**Interaction of GPHR and TPP1F.** (A) Immunofluorescence analysis of AX2 cells expressing GPHR-GFP. Methanol-fixed cells stained with vatA-specific antibodies (top panel) and TPP1F-specific mAb K88.254-3 (bottom panel). Nuclei are labeled with DAPI (merge, in blue). Scale bar: 10 µm. (B) GPHR-GFP is precipitated by GST-TPP1F (left). GPHR-GFP, whole-cell lysate of GPHR^−^ cells expressing GPHR-GFP (4×10^5^ cells). Pull down was carried out with GST and GST-TPP1F carrying glutathione-Sepharose beads as indicated (pull down). The blot was probed with mAb K3-184-2 (WB: GFP). Right panel shows endogenous TPP1F precipitated by GPHR-GFP. AX2 cell lysates expressing GPHR-GFP were used for the pull down with GFP-trap beads. TPP1F was detected with mAb K88-254-3. AX2 cells expressing phr2ab-GFP, phosphatase 2A regulatory B subunit, with similar apparent molecular size as GPHR, were used as a control. (C) GST-DUF3735 interacts with TPP1F-GFP. TPP1F-GFP, whole-cell lysate. The pull down was carried out with GST and GST-DUF3735 (pull down). The blot was probed with mAb K3-184-2 (WB: GFP). (D) Identification of the GPHR binding domain in TPP1F. GST, GST-prodomain and GST-catalytic domain of TPP1F bound to glutathione-Sepharose beads were used to pull down GPHR-GFP. The blot was probed with mAb K3-184-2. (E) TPP1 activity in lysates obtained from various strains as indicated. Lysates were prepared from equal numbers of cells and equal volumes were used in the assay. TPP1 activity is given as activity as a percentage of AX2 value (set to 100%). Mean±s.d. values from 4 experiments are shown (*P<0.05; **P<0.01; ***P<0.001). (F) TPP1F levels in AX2 and GPHR^−^. Whole-cell lysates (4×10^5^ cells) were separated by SDS-PAGE (10% acrylamide). The blot was probed with mAb K88-254-3. cap34 detected by mAb 135-409 was used to assess loading. (G) TPP1F-specific transcript amounts in AX2 expressing TPP1F-GFP and GPHR^−^ expressing TPP1F-GFP as quantified by qRT-PCR. Normalization was done using GAPDH. The value obtained for AX2 was set to 100%. The data represent mean±s.d. values from three experiments (***P<0.001). (H) Expression of TPP1F-GFP in GPHR^−^. Whole-cell lysates of GPHR^−^ expressing TPP1F-GFP and GPHR^−^ probed with mAb K3-184-2. (I) Comparison of TPP1F-GFP and vatA distribution in GPHR^−^ and AX2. TPP1F-GFP was detected with polyclonal antibodies specific for GFP, vatA with mAb 221-35-2. Nuclei are stained with DAPI (merge, in blue). Scale bar: 10 µm. (J) Interaction of GST-TPP1B with GPHR-GFP. GST and GST-TPP1B bound to glutathione-Sepharose beads were used for the pull down from AX2 cells expressing GPHR-GFP. The precipitate was probed with mAb K3-184-2. The images are derived from a single blot; however, a shorter exposure is shown for GPHR-GFP.

Since TPP1F was present in the endo-lysosomal compartment, we re-evaluated the localization of its binding partner GPHR in AX2 cells and performed co-immunofluorescence studies with anti-vatA antibodies. We in fact observed GPHR-GFP also in this compartment (Fig. 4A), revealing that GPHR is present in subcellular membranous compartments reaching from ER membranes to membranes of the endo-lysosomal system. Staining of GPHR-GFP-expressing cells with mAb K88-254-3 for TPP1F indicated some overlap but was less pronounced (Fig. 4A).

In further experiments we tried to obtain independent proof of the GPHR and TPP1F interaction and found that GST-TPP1F could pull down GPHR-GFP from AX2 cells (Fig. 4B, left panel). In the reverse experiment, we precipitated GPHR-GFP from AX2 cells and detected endogenous TPP1F in the precipitate with mAb K88-254-3 (Fig. 4B, right panel). We also used a recombinant GPHR-derived polypeptide (residues 174-270) expressed as a GST fusion protein for pull down. This polypeptide encompassed the DUF3735 domain (residues 192-247) of GPHR and was designated GST-DUF3735. According to the membrane protein topology prediction program TOPCONS (topcons.cbr.su.se), this region should face the lumen of the vesicles. GST-DUF3735 precipitated TPP1F-GFP, assigning a role to the DUF domain as interaction domain (Fig. 4C). GST used for control did not interact with TPP1F. We then used GST-DUF3735 in TPP1 activity assays and found that upon its addition, the TPP1 activity was reduced to background levels. Addition of comparable amounts of GST to the assay did not have an effect on the activity (Fig. 1D). Using the GST-prodomain and GST-catalytic domain of TPP1F for pull down of GPHR-GFP, only GST-prodomain could pull down GPHR-GFP, localizing the binding site to the prodomain. GST alone did not bring down GPHR-GFP (Fig. 4D).

Next, we compared the TPP1 activity of AX2 wild-type cells and various mutant strains as described in the Materials and Methods section. Lysates from equal cell numbers were prepared and aliquots used. The activity of AX2 was taken for reference and set to 100%. In GPHR^−^, the activity was reduced to ∼50-60% of AX2 values (Fig. 4E). The result was corroborated by western blots where we observed reduced TPP1F protein levels in GPHR^−^ as compared to AX2 (Fig. 4F). TPP1F-GFP expressing AX2 and GPHR^−^ cells showed increased activities as compared to AX2 (>120% and ∼150%, respectively) (Fig. 4E). Consistent with this result, quantification of the *tpp1F* mRNA levels by qRT-PCR analysis in AX2 and GPHR^−^ cells expressing TPP1-GFP revealed higher levels in the GPHR^−^ strain (Fig. 4G).

We studied TPP1F-GFP in the absence of its interaction partner in GPHR^−^ cells and found that the protein was expressed as in AX2 with two bands of ∼95 and 120 kDa (Fig. 4H). Immunofluorescence analysis showed a similar distribution and some colocalization with vatA-stained structures (Fig. 4I). From our results, we conclude that GPHR can interfere with TPP1F activity. Furthermore, it may not be required for correct trafficking of TPP1F but seems to be essential for its levels since we observed a reduced activity as well as reduced protein amounts in GPHR^−^ cells (Fig. 4E,F).

### *D. discoideum* harbors at least seven genes encoding tripeptidyl peptidase 1

Using the ddTPP1 sequence (DDB_G0269914) reported previously (Phillips and Gomer, 2015), we analyzed the *D. discoideum* genome (Eichinger et al., 2005; http://dictybase.org/) and found eight sequences with high homologies to tripeptidyl peptidases 1. The proteins fell into two groups. The members of group 1 (TPP1A-C) resembled ddTPP1 (now TPP1A) and had the same overall structure as mammalian TPP1. TPP1D-TPP1F belonged to the second group and resembled TPP1F (also known as v4-7). In all three proteins, the peptidase domain is interrupted in its central region by an insert of more than 120 amino acids, for which no structural prediction is available (Fig. S1). The inserts are conserved and share more than 30% identical and more than 60% similar residues. All TPP1 enzymes with the exception of TPP1F carry several potential N-glycosylation sites distributed throughout the proteins.

Of the other two proteins, DDB_G0272028, a member of group 2, is a pseudogene (tpp1_ps), and DDB_G0291952 is composed of the protease prodomain only (Fig. S1). In general, the N-terminal prodomains in TPP1 enzymes have chaperone activity and function as intramolecular chaperones required for the folding of the catalytic domains. Interestingly, in *Aeromonas sobria*, a separate pro-fragment encoding gene has been identified, which associates with a serine protease that lacks a propeptidase (Kobayashi et al., 2015). We have not detected a serine protease lacking the propeptidase in the *D*. *discoideum* genome. However, if its amino acid sequence is extremely divergent it will have escaped our notice.

Next, we collected expression data for the seven genes from publicly available RNA-seq data (Parikh et al., 2010; Schilde et al., 2016; see Materials and Methods). For all genes, with the exception of *tpp1F* we observed remarkable changes during development. Furthermore, the transcript abundance varied greatly. The highest transcript levels throughout growth and development were observed for *tpp1B* and *tpp1F*. All other genes were expressed at significantly lower levels, in particular, *tpp1D* and *tpp1E* showed very low mRNA levels. For the gene encoding the protease prodomain only, moderate expression was observed only during late development (Fig. S2).

Based on the presence of several TPP1 enzymes in *D. discoideum*, we wondered whether they can also interact with GPHR and expressed TPP1B (DBB0306176), the most abundant enzyme during growth according to transcript levels, as a GST-fusion and used it for pull down of GPHR-GFP. We in fact successfully precipitated the protein, which makes our proposal for a link between TPP1 and GPHR more general (Fig. 4J).

### TPP1F-knockout cells do not display an obvious phenotype

**Fig. 5.**
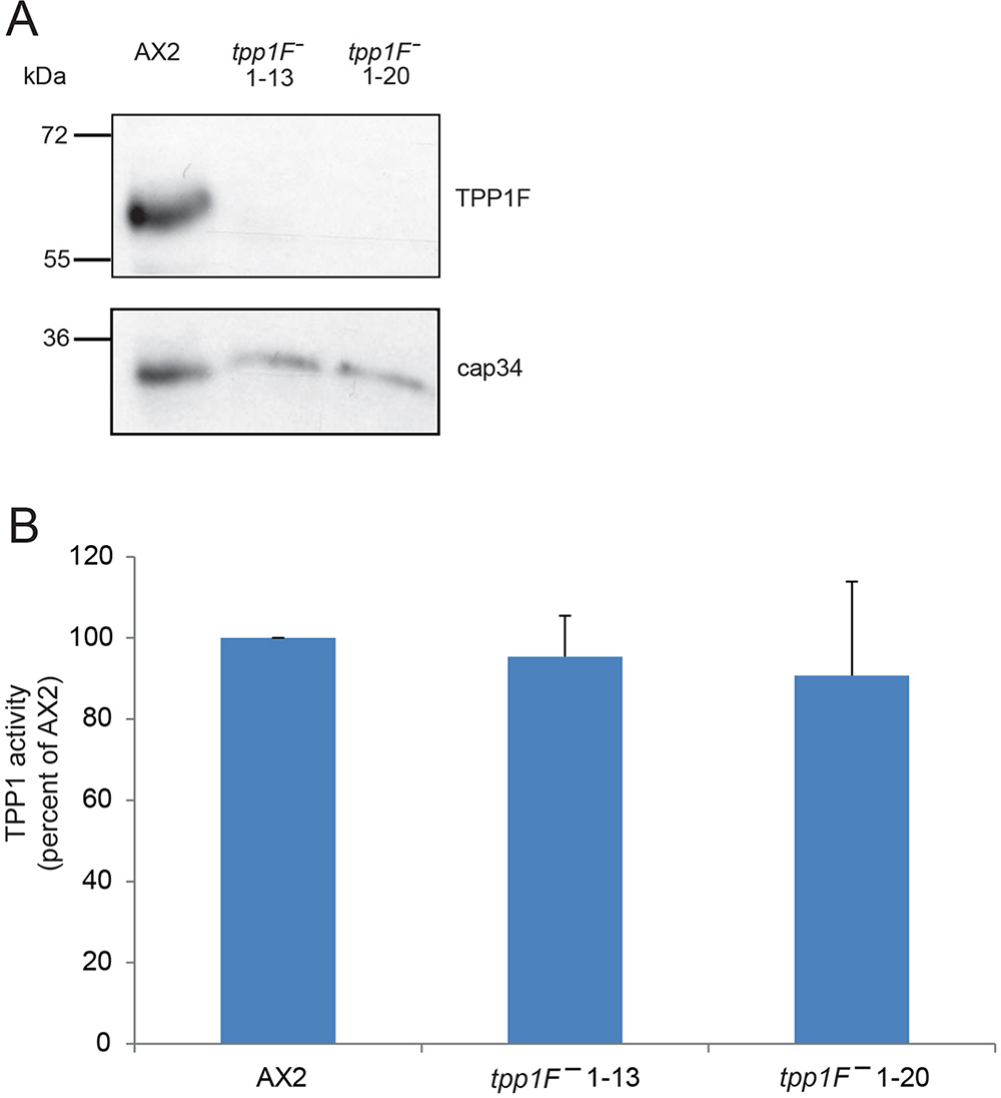
**Characterization of TPP1F-deficient cells.** (A) Mutant 1-13 and 1-20 do not express the ∼65 kDa protein as revealed by analysis of whole-cell lysates (5×10^5^ cell equivalents per lane) with mAb K88-254-3 (TPP1F). Loading was assessed using cap34-specific mAb 135-409. (B) TPP1 activity is not impaired in growing mutant cells. Lysates from equal numbers of cells were used in the TPP1 assay. The data are the mean±s.d. from four independent experiments. The results from AX2 were set to 100%. The differences were not statistically significant.

TPP1F-deficient cells were generated by homologous recombination. The knockout was identified by PCR analysis using appropriate primers and confirmed by western blotting, where incubation with mAb K88-254-3 revealed the absence of the protein in whole-cell homogenates (Fig. 5A; Fig. S3A). We obtained several independent knockout strains. Clones 1-13 and 1-20 were used in all further studies. In immunofluorescence analysis, staining with mAb K88-164-8 was abrogated (see Fig. 2D, shown for clone 1-13). TPP1 assays using cell lysates from the knockout lines showed similar activity levels to that in the wild type (Fig. 5B). The observed activity in growing cells is presumably due to TPP1B since *tpp1B* transcript levels are the highest of all *D. discoideum* TPP1 genes during growth (∼2400 rpkm compared with ∼630 rpkm for *tpp1F*; Fig. S2). Growth on a lawn of *Klebsiella* and in shaking suspension was not altered. Cell substratum adhesion was not impaired, development in shaking suspension occurred timely, and during development on a substratum, the knockout strains formed normal-looking fruiting bodies containing oval spores as in the AX2 wild type. Slug formation was not altered and the slugs were phototactically active. The spores could be stained with Calcofluor, which further indicates that development and differentiation was not grossly affected, and they germinated in a similar manner to AX2 spores (Fig. S3B and data not shown).

## DISCUSSION

Tripeptidyl peptidase 1 sequences have been identified in the genomes of mammals, fish and amphibians, but they are not found in *Drosophila*, *Caenorhabditis elegans* and *Saccharomyces cerevisiae* (Wlodawer et al., 2003). Remarkably, *D. discoideum* harbors at least six genes coding for functional proteins, among them *tpp1F*/*v4-7*, which is described here. This fairly unique situation could be due to the lifestyle of *D. discoideum* as a natural phagocyte. Furthermore, TPP1D-TPP1F are unusual in so far as their peptidases S53 domain is interrupted by a stretch of about 120 amino acids, which is highly conserved among the proteins. The interruption of the catalytic domain is, however, not unique to *D. discoideum* TPP1D-TPP1F. Similar protein structures have been found in the genomes of other Dictyostelidae, three enzymes in *D. purpureum* and *D. lacteum*, and one each in *Polysphondylium pallidum*, *D. fasciculatum* and *Acytostelium subglobosum* (Sucgang et al., 2011; Heidel et al., 2011; Urushihara et al., 2015). Furthermore, the flagellate *Thecoamonas trahens* and the filamentous brown algae *Ectocarpus siliculosis* harbor similar enzymes (XP_013754467; CBJ33603.1). In these proteins, the sequences that interrupt the peptidases S53 domain exhibit 38-56% identity and 54-73% similarity with the sequence of TPP1F. In addition, a related sequence was detected in a P-loop-containing nucleoside triphosphate hydrolase of *Thiomargarita nelsonii* (KHD09205), a sulfide-oxidizing marine bacterium.

TPP1F has been found previously in a secreted proteome (Bakthavatsalam and Gomer, 2010). In this work, the authors identified by mass spectrometry 349 proteins secreted by developing *D. discoideum* AX2 cells. They found known secreted proteins, proteins secreted in multivesicular bodies such as adenylyl cyclase, other signaling proteins and quorum-sensing proteins. TPP1F, was also detected throughout development. We identified TPP1F as binding partner of GPHR, a protein associated with membranes of the ER, Golgi and endo-lysosomal membranes, and with roles in phagocytosis, secretion, growth and development in *D. discoideum* (Deckstein et al., 2015). TPP1F interacted with the DUF3735 domain, recently renamed as GPHR_N domain (pfam 12537). This domain has been found in eukaryotic proteins. It contains the fifth transmembrane domain surrounded by the neighboring loop regions of GPHR and is ∼60-70 amino acids in length. So far, no particular function has been assigned to it. The function of the GPHR-TPP1F interaction is also unknown. It might well be that it is important for several aspects of TPP1 or GPHR biology. Thus, it could help to transport TPP1F, a soluble lumenal protein, to its final destination. Alternatively, it could be that GPHR, which was identified as pH regulator in mammalian cells, contributes to the establishment of the low pH required for TPP1 activation and activity. The interaction could also be involved in the degradation of GPHR in the lysosome. We have indications from *in vitro* activity measurements that the TPP1F activity could be inhibited by the recombinant DUF3735 polypeptide of GPHR. Our attempts to show an interaction for the orthologous mammalian proteins were not successful because the commercially available antibodies were not useful in these experiments.

CLN2 is a devastating disease, for which no cure is currently available. By examining the mutations that resulted in disease, it was found that they were distributed over the length of the gene and protein and affected the prodomain as well as the peptidase domain. They represented missense mutations and led to premature termination or splicing defects (Kousi et al., 2012). So far, no mutations in the *GPR89A* and *-B* genes have been identified that were linked to human disease (Peterson et al., 2010). However, in mouse, a loss of GPHR was embryonic lethal, and a tissue-specific knockout led to an impairment of the skin barrier (Tarutani et al., 2012).

TPP1F is the second tripeptidyl peptidase 1 described in *D. discoideum*. The first enzyme, TPP1A, was reported by Phillips and Gomer (2015) who showed that it was an ortholog of human TPP1. *tpp1A* transcripts are present at very low levels during the growth phase and strongly increased during development, reaching high levels in later development, particularly in prespore cells. Mutant cells, in which the gene had been disrupted, showed precocious development, reduced spore formation and reduced viability under autophagy-inducing conditions. Tripeptidyl peptidase 1 activity was reduced but not abrogated compared with wild-type cells. This is quite plausible in view of the presence of several TPP1-encoding genes and the findings that *tpp1B* and *tpp1F* in particular, are expressed throughout development and their transcripts are present at significantly higher levels than those of *tpp1A* (Fig. S2). Based on the expression pattern, TPP1A might contribute to the total activity to a significant degree only during the later stages of development (t16) and in a cell-type-specific manner, as mutations in the corresponding gene lead to reduced spore formation (Phillips and Gomer, 2015).

In conclusion, we show here that *D. discoideum* harbors several TPP1-encoding genes, which are expressed in a development-specific manner. This situation is fairly unique since mammals have only a single TPP1 gene in their genomes. Furthermore, the *Dictyostelium* proteins could be divided into two groups, which is another unique feature. The group comprising TPP1A-TPP1C had a structure similar to the mammalian proteins, whereas in the enzymes of the second group, the catalytic domain was interrupted by an insert. The role of this insert is unknown. We further revealed a new and unexpected connection between TPP1 and GPHR, a highly conserved component of intracellular membranes. The interaction was observed for enzymes of both groups and took place between the prodomain of TPP1 and the DUF3735 domain of GPHR. These domains are also present in mammalian TPP1 and GPHR and therefore a similar interaction might take place and GPHR could interfere with TPP1 activity. Whether this interaction is important for the disease pathology needs to be explored. Since the majority of the TPP1 mutations in NCL2 result in the reduction or loss of enzyme activity, we may have found a tool with which one can interfere in the disease progression (Wlodawer et al., 2003; Kousi et al., 2012).

Based on these findings, the potential of *D. discoideum* to contribute to the study of the functions of proteins linked to neuronal ceroid lipofuscinosis is strengthened and can lead to new treatment options (Huber, 2016).

## MATERIALS AND METHODS

### Growth and transformation of *D. discoideum*

Wild-type strain AX2 and GPHR^−^ were grown at 22°C as described (Deckstein et al., 2015). AX2 and GPHR transformants expressing TPP1F-GFP were selected with G418 (2 µg/ml) and identified by western blotting of whole-cell lysates. AX2 expressing Nramp1-GFP is described in Sultana et al. (2005).

### Expression of recombinant protein in bacteria, generation of knockout mutants and antibody generation

The v4-7 sequences were amplified from cDNA. Residues corresponding to amino acids 1-702 were cloned into pDex79 (Westphal et al., 1997) to obtain TPP1F-GFP. The GFP-tag was present at the C-terminus of TPP1F. For expression as full-length recombinant protein in *E. coli* Arctic Express, sequences corresponding to amino acids 21-702 lacking the signal peptide were cloned into pGEX4T (GE Healthcare). For expression of the prodomain, amino acid residues 21-200, and for expression of the catalytic domain, residues 201-702 were expressed in pGEX4T. GST was present at the N-terminus of the proteins. For expression of full-length TPP1B as a GST-fusion protein, sequences corresponding to amino acid residues 21-598 were cloned into pGEX4T1. For generation of TPP1F-knockout cells, a gene replacement vector was generated and cDNA sequences 192-690 and 1452-1952 were cloned into plasmid pLPBLP (Faix et al., 2004). Selection of transformants was with blasticidin (1.5 µg/µl). Single colonies were analyzed by PCR using appropriate primers specific for the homologous recombination event (see Fig. S3). Confirmation was by western blot analysis using TPP1F-specific monoclonal antibodies.

For generation of monoclonal antibodies, recombinant GST-TPP1F fusion protein (residues 20-702) was used for immunization of BALB/c mice as described (Schleicher et al., 1984). Hybridomas were screened for their ability to recognize the TPP1F-GFP fusion protein in AX2 transformants and the endogenous protein in wild-type AX2 in western blots and by immunofluorescence analysis. K88-7-2, K88-254-6 and K88-164-8 mAbs were used. All antibodies isolated could be used for western blot and immunofluorescence analysis and gave similar results. Mice were handled in accordance with the German Animal Welfare Act (Tierschutzgesetz) as well as the German Regulation for the protection of animals used for experimental purposes or other scientific purposes (Tierschutz-Versuchstierverordnung), and the investigations were approved by the responsible governmental animal care and use office (Landesamt für Natur, Umwelt und Verbraucherschutz North Rhine-Westphalia, Recklinghausen, Germany; reference number 84-02.05.40.14.080).

For amplification of the DUF3735 domain of GPHR, forward 5′-GGATCCGGTTTAGTTTCTTTAGAAATGGGTATT-3′ and reverse 5′-GAATTCTCTTTTTTTTTTATTAAAAATTTTATCTAAAGCATG-3′ primers were used. *Bam*HI and *Eco*RI sequences were added to the 5′ and 3′ ends for cloning into pGEX4T-1. For protein expression, Arctic Express cells were used. GST-DUF3735 was the only GPHR-derived polypeptide, which could be expressed in sufficient amounts in *E. coli*.

### Mutant analysis

Mutant cells were grown on *Klebsiella* plates and in axenic medium. Growth was analyzed for both conditions. Development was assayed on phosphate agar plates (5×10^7^ cells per 10 cm dish) and in suspension in Sørensen phosphate buffer (17 mM sodium potassium phosphate, pH 6.0) at a density of 1×10^7^ cells/ml and shaking at 160 rpm. Cell substratum adhesion, phototaxis and spore germination analysis were performed as described (Deckstein et al., 2015). Spores were stained with Calcofluor White stain (Fluka). Spore size and shape were assessed after staining with Calcofluor. Spores were incubated for 1 min in the solution prepared according to the data sheet and analyzed under UV.

### RNA-seq data generation and quantitative RT-PCR analysis

The RNA-seq raw data were obtained from a previous study (AX4 strain two replicates; Parikh et al., 2010) and the NC4 data from water agar developmental time points (one experiment). The NC4 RNA-seq data were published in Schilde et al. (2016). These data were combined with the RNA-seq data from Parikh et al. (2010). Only time points concurrent between the two data sets were used. This way we ensured that the expression profiles could be calculated from independent biological experiments. The NC4 developmental time course is more variable than that obtained with axenically grown AX4. Thus, the NC4 RNA samples were taken at distinct developmental stages closely matching the AX4 time points. To make the data comparable across experiments, we normalized all data to rpkm values (reads per kb per million mapped reads; see Mortazavi et al., 2008).

Quantitative RT-PCR analysis was performed as described (Deckstein et al., 2015). For TPP1F analysis, primers TPP1F-192-for, AGAAGAGAAATTCTGGGATGTTTCATCACC and TPP1F-690-rev, GGCAGCAATTGATTGGAAGTTTTCTGATTC were used. The conditions were 15 min at 95°C (initial step), followed by 41 cycles of 30 s 94°C, 45 s 57°C, 40 s 68°C in a 25 µl volume. As quantification standard, defined concentrations of annexinA7 cDNA were used. GAPDH levels were used for normalization.

### Pull-down experiments and immunoprecipitation

For pull-down experiments, GST-tagged proteins were expressed in *E. coli* XL1blue (GST-prodomain) or *E. coli* Arctic Express (all other fusion proteins) as recommended by the manufacturer (Agilent Technologies). Induction of expression was with 0.1 mM isopropyl-β-D-thiogalactopyranoside (IPTG). Cells were lysed and the protein in the supernatant bound to glutathione Sepharose 4B beads (GE Healthcare). For GST-TPP1F, the protein was solubilized from the cell pellet by addition of sodium phosphate buffer (20 mM, pH 6.5) as described for the purification of a bacterial enzyme (Oda et al., 1994). Fusion proteins bound to beads, as well as GST bound to beads for the control, were used to pull down GFP-tagged proteins from *D. discoideum* strains. To obtain soluble GPHR-GFP and TTP1-GFP, the *D. discoideum* cells were homogenized in lysis buffer (10 mM triethanolamine, 10 mM acetic acid, 1 mM EDTA, 250 mM sucrose, pH 7.4) as described (Shina et al., 2010). For pull-down and immunoprecipitation (IP) experiments, Tris-HCl (final concentration 10 mM), pH 7.5, and NaCl (final concentration 100 mM) were added to the lysate. For IP, Protein A-Sepharose (Sigma-Aldrich)-bound GFP-specific monoclonal (mAb K3-184-2) or polyclonal (pAb) antibodies were used (Noegel et al., 2004). Alternatively, GFP-Trap beads (ChromoTek, Planegg-Martinsried, Germany) were used to pull down GFP-tagged proteins. The proteins were eluted from the beads using SDS sample buffer, separated by SDS-PAGE (10-12% acrylamide), immunoblotted and probed with mAb K3-184-2.

For identification of GPHR binding partners, AX2 cells expressing GPHR-GFP were used. AX2 cells expressing GFP served as control. In a typical experiment, 1×10^8^ cells were lysed as described above. For immunoprecipitation, Protein A-Sepharose carrying polyclonal GFP antibodies were used. The beads were washed with lysis buffer without sucrose, the proteins eluted from the beads, separated by SDS-PAGE (10% acrylamide), stained with Coomassie Blue, and bands in the correct molecular mass range cut out and subjected to LCMS analysis.

### Immunofluorescence, antibodies and western blot analysis

Methanol-fixed cells were stained with mAb K3-184-2 or rabbit polyclonal antibodies specific for GFP (Noegel et al., 2004), rabbit polyclonal antibodies specific for DdLIMP (Karakesisoglou et al., 1999), mAb 221-64-1 recognizing protein disulfide isomerase (PDI) (Monnat et al., 1997), vatA-specific mAb 221-35-2 (Jenne et al., 1998), mAb 70-100-1 labeling mitochondria (Troll et al., 1992) and with the Golgi-specific mAb 190-340-8 (Weiner et al., 1993). DNA was stained with DAPI. Detection was with Alexa Fluor 568- or 488- or Cy3-labeled secondary anti-mouse or anti rabbit IgG. For western blotting, proteins were separated in SDS-polyacrylamide gels (10 and 12% acrylamide), transferred to nitrocellulose membranes and probed with the appropriate antibodies. Proteins were detected with enhanced chemiluminescence. Hybridoma supernatants (appropriately diluted) were used in the experiments. For loading controls, cap34-specific mAb 135-409 was used (Hartmann et al., 1989).

### Enzyme assays

For TPP1 activity measurements, an enzymatic assay using Ala-Ala-Phe 7-amido-4-methylcoumarin (Sigma-Aldrich) as fluorescent substrate was used. TPP1-GFP and whole-cell lysates were tested. TPP1-GFP was precipitated by polyclonal GFP-specific antibodies from lysates of AX2 cells expressing the fusion protein and the precipitate used in the enzyme assay (Sohar et al., 2000). For inhibition assays, equal amounts (1 µg per assay) of recombinant GST-DU3735 and GST were added to the assay. For enzyme assays involving various *D*. *discoideum* strains, whole-cell lysates were prepared from equal numbers of cells. Cells were lysed in 50 mM sodium phosphate buffer, pH 6.5, and 0.5% NP40 (4×10^6^ cells/100 µl); 20 µl of the lysate was used per assay. Lysis was controlled microscopically. As a positive control, blood derived from mice, spotted onto filter paper and dried was used (dried blood spot, DBS) (Lukacs et al., 2003). The TPP1 reaction buffer contained the substrate (250 µM), 150 mM NaCl, 0.1% Triton X-100, 100 mM sodium actetate, pH 4.5. Incubation was at 37°C in the dark for 1 h. The reaction was terminated by addition of stop solution (100 µl per 100 µl assay volume). The stop solution contained 100 mM monochloroacetic acid, 130 mM NaOH, 100 mM acetic acid, pH 4.3. The fluorescent peptide released was quantified in a plate reader (Tecan Infinite M1000) using excitation at 360 nm and emission at 460 nm. All reagents were from Sigma. For statistical evaluation, analysis of variance (ANOVA) was carried out. The data presented are derived from between three and five independent experiments.
